# Characteristics of brain glucose metabolism and metabolic connectivity in noise-induced hearing loss

**DOI:** 10.1038/s41598-023-48911-x

**Published:** 2023-12-11

**Authors:** Seunghyeon Shin, Hyun-Yeol Nam

**Affiliations:** Department of Nuclear Medicine, Samsung Changwon Hospital, Sungkyunkwan University School of Medicine, Changwon, Republic of Korea

**Keywords:** Neuroscience, Auditory system, Cortex

## Abstract

The purpose of this study was to evaluate the differences in cerebral glucose metabolism and metabolic connectivity between noise-induced hearing loss (NIHL) subjects and normal subjects. Eighty-nine subjects who needed close observation for NIHL or were diagnosed with NIHL and 89 normal subjects were enrolled. After pre-processing of positron emission tomography images including co-registration, spatial normalization, and smoothing, a two-sample t-test was conducted to compare cerebral glucose metabolism between the two groups. To evaluate metabolic connectivity between two groups, BRAPH–BRain Analysis using graPH theory, a software package to perform graph theory analysis of the brain connectome was used. NIHL subjects showed hypometabolism compared to normal subjects in both insulae (x − 38, y − 18, z 4; × 42, y − 12, z 4) and right superior temporal gyrus (× 44, y 16, z − 20). No brain regions showed hypermetabolism in the NIHL subjects. In metabolic connectivity analysis, NIHL subjects showed decreased average strength, global efficiency, local efficiency, and mean clustering coefficient when compared with normal subjects. Decreased glucose metabolism and metabolic connectivity in NIHL subject might reflect decreased auditory function. It might be characteristic of sensorineural hearing loss.

## Introduction

Hearing loss is the fourth leading cause of disability and impairs communication with adverse effects on relationships with other people^1^. Hearing loss is defined by the audiometric threshold^2^. The World Health Organization (WHO) has classified hearing impairment according to pure-tone audiometry in the better hearing ear as: (1) no impairment, ≤ 25 dB hearing threshold level (HL); (2) slight impairment, 26–40 dB HL; (3) moderate impairment, 41–60 dB HL; (4) severe impairment, 61–80 dB HL; and (5) profound impairment, ≥ 81 dB HL^3^. Peripheral hearing loss is categorized as conductive or sensorineural hearing loss^1^. Conductive hearing loss is caused by impairment of the outer or middle ear, while sensorineural hearing loss is caused by the dysfunction of the cochlea or spiral ganglion^1^.

Noise-induced hearing loss (NIHL) is a sensorineural hearing loss that affects the hair cells of the inner ear. This is typically symmetric^4^. Noise exposure below 70 dB is considered safe sound level^5^. Repeated or prolonged noise exposure above 70 dB can result in NIHL over time^5^. Noise above 120 dB could cause immediate and permanent hearing loss^5^. Noise damages the hair cells of the inner ear by causing direct mechanical stress with intense sound pressure and by causing metabolic damage via free radicals or reactive oxygen species^1,6^. Moreover, free radicals can lead to vasoconstriction and reperfusion of cochlear cell resulting in cell death^5^. After noise exposure, pro-inflammatory cytokines including tumor necrosis factor-alpha (TNF-α), interleukins, and chemokines are induced in cochlear^5^. Some of these cytokines are known to have ototoxicity^5^. Excessive release of glutamate resulting in glutamate excitotoxicity is also caused by noise exposure^5^. Early or moderately advanced NIHL typically shows a notch at 4 kHz with a range of 3 kHz to 6 kHz in a pure tone audiogram^6^. Chronic noise exposure can make the notch deeper and wider, eventually involving lower frequencies, including 0.5, 1, and 2 kHz^6^.

2-Deoxy-2-[fluorine-18]fluoro-d-glucose (F-18 FDG) positron emission tomography (PET)/computed tomography (CT) is widely used in oncology^7^. Since F-18 FDG shows cerebral glucose metabolism, which reflects neuronal and glial activity, F-18 FDG PET/CT is also widely used in neurology and psychiatry^8^. Previous studies have evaluated the correlation between cerebral glucose metabolism and hearing loss. Although there are some differences between studies, most studies have reported that glucose metabolism is decreased in the auditory cortex^9–13^. However, the correlation between cerebral glucose metabolism and NIHL has not yet been reported. Although NIHL is a type of sensorineural hearing loss, studies enrolling hearing loss subjects with the same cause have not yet been reported. Previous studies have mainly evaluated the cerebral glucose metabolism in subjects with profound hearing loss^9–12^. Thus, revealing the relationship between hearing loss and cerebral glucose metabolism in NIHL subjects might provide new information on changes in cerebral glucose metabolism in subjects with hearing loss.

Brain connectivity is divided into structural and functional connectivity^14^. Structural connectivity is defined as the anatomical organization of the brain by means of fiber tracts, and functional connectivity is defined as the statistical dependence between the time series of electrophysiological activity and (de)oxygenated blood levels in distinct regions of the brain^14^. Functional connectivity studies have been conducted using functional magnetic resonance imaging (MRI), electroencephalography, and magnetoencephalography^14^. However, metabolic connectivity, which is defined as relationships between metabolic measurements in different brain regions, is emerging^15^. Several studies have reported altered metabolic connectivity in subjects with hearing loss^10,16,17^. However, a metabolic connectivity study was not performed in subjects with NIHL. Thus, the objective of this study was to determine whether cerebral glucose metabolism and metabolic connectivity in subjects with NIHL might be different from those in normal subjects.

## Results

### Characteristics in NIHL and normal subjects

**Figure 1:**
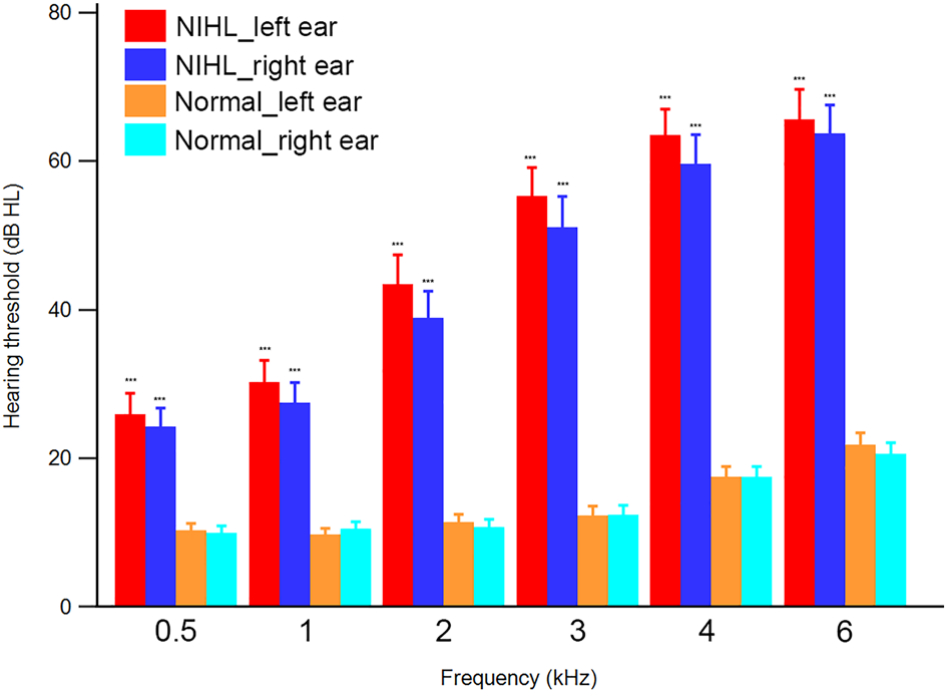
The differences of hearing thresholds between the NIHL and normal subjects at 0.5, 1, 2, 3, 4 and 6 kHz. ***p < 0.001 (NIHL vs normal subject). *NIHL* noise-induced hearing loss.

A total of 178 male subjects (89 NIHL subjects and 89 normal subjects) were included in this study. The characteristics of NIHL and normal subjects are summarized in Table 1. Pure tone audiometry mean values (left/right) of NIHL subjects were 25.7 ± 14.4/24.0 ± 13.2 at 0.5 kHz, 30.1 ± 15.1/27.2 ± 14.1 at 1 kHz, 43.3 ± 19.9/38.7 ± 18.1 at 2 kHz, 55.1 ± 19.3/51.0 ± 20.7 at 3 kHz, 63.3 ± 17.8/59.4 ± 19.9 at 4 kHz, 65.4 ± 20.5/63.5 ± 19.6 at 6 kHz. Those of normal subjects were 10.1 ± 5.5/9.7 ± 5.8, 9.6 ± 5.1/10.3 ± 5.4, 11.1 ± 6.2/10.5 ± 6.4, 12.0 ± 7.8/12.1 ± 7.7, 17.3 ± 7.9/17.2 ± 8.3, 21.6 ± 8.7/20.3 ± 8.4, respectively. There was no significant difference between left and right values of pure tone audiometry. All values of left and right pure tone audiometry in NIHL subjects were significantly higher than those of normal subjects (P < 0.001). Figure 1 showed the differences of hearing thresholds between the NIHL and normal subjects at 0.5, 1, 2, 3, 4 and 6 kHz. When the NIHL subjects were categorized by WHO classification, 21 subjects had no impairment, 46 subjects had slight impairment, 21 subjects had moderate impairment, and one subject had severe impairment.

**Table 1:** Subjects characteristics.

Variables	NIHL subjects (n = 89)	Normal subjects (n = 89)
Age (years)	50.1 ± 6.3	50.1 ± 6.3
Gender (male/female)	89/0	89/0
Fasting glucose (mg/dL)	96.9 ± 21.2	94.9 ± 12.3
HbA1C (%)	5.7 ± 0.9	5.5 ± 0.4
BMI	24.4 ± 2.8	24.6 ± 2.8
Pure tone averages (left/right)*	33.0 ± 13.5/30.0 ± 12.3	10.3 ± 4.4/10.2 ± 4.4

Data are mean ± SD values. Pure tone average = (0.5 kHz + 1 kHz + 2 kHz)/3.

*NIHL* noise-induced hearing loss, *HbA1C* glycated haemoglobin, *BMI* body mass index.

*p < 0.001.

### Difference in brain glucose metabolism between NIHL and normal subjects

**Figure 2:**
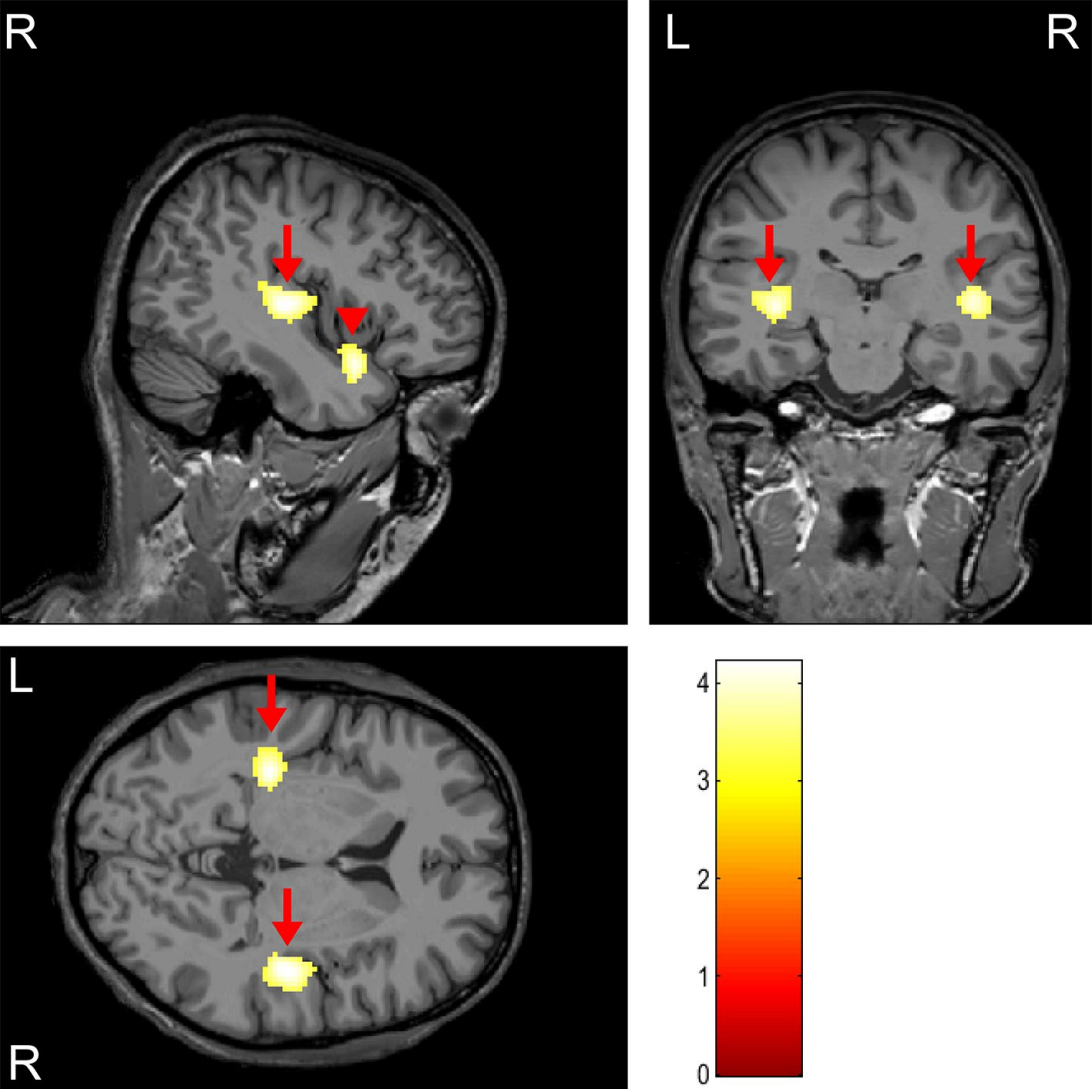
Brain areas showing hypometabolism in noise-induced hearing loss subjects (arrow: insula; arrow head: right superior temporal gyrus).

NIHL subjects showed hypometabolism than normal subjects in both insulae (x − 38, y − 18, z 4; × 42, y − 12, z 4) and right superior temporal gyrus (× 44, y 16, z − 20) (Fig. 2 and Table 2). No brain regions showed hypermetabolism in the NIHL subjects.

**Table 2:** Brain regions showing hypometabolism in NIHL subjects.

Brain area	Brodmann area	Coordinates (x, y, z)	Voxels	p (uncorrected)	T
Left hemisphere					
Insula	13	− 38, − 18, 4	289	p < 0.001	4.17
Right hemisphere					
Insula	13	42, − 12, 4	301	P < 0.001	4.21
Superior temporal gyrus	38	44, 16, − 20	115	P < 0.001	4.13

*NIHL* noise-induced hearing loss.

### Difference in metabolic connectivity between NIHL and normal subjects

In terms of global connectivity, NIHL subjects showed decreased average strength, global efficiency, local efficiency, and mean clustering coefficient when compared with normal subjects (45.0222 vs. 46.2889, p = 0.0300; 0.2072 vs. 0.2254, p = 0.0460; 0.4541 vs. 0.5842, p = 0.0230; 0.1290 vs. 0.1722, p = 0.0070, respectively) (Table 3).

**Table 3:** The difference of global metabolic connectivity in NIHL and normal subjects.

	NIHL subjects	Normal subjects	Difference	p
Average degree	45.0222	46.2889	1.2667	0.4160
Average strength	9.7358	11.4143	1.6784	0.0300*
Characteristic path length	5.9285	5.5715	− 0.3570	0.0820
Global efficiency	0.2072	0.2254	0.0181	0.0460*
Local efficiency	0.4541	0.5842	0.1301	0.0230*
Mean clustering coefficient	0.1290	0.1722	0.0432	0.0070*
Transitivity	0.1915	0.2388	0.0473	0.0540
Modularity	0.3632	0.3989	0.0357	0.1990
Assortative coefficient	0.1753	0.1028	− 0.0725	0.2610
Small-worldness	0.7888	0.8504	0.0616	0.0750

*NIHL* noise-induced hearing loss.

*p < 0.05.

In terms of nodal connectivity, local efficiency and clustering coefficiency of the left insula were significantly lower in NIHL subjects than in normal subjects. In the right insula, strength, global efficiency, local efficiency, and clustering coefficiency were significantly lower in subjects with NIHL. In the right superior temporal gyrus, strength, global efficiency, local efficiency, and closeness centrality were significantly lower, and path length was significantly higher in NIHL subjects. Detailed results are presented in Table 4.

**Table 4:** The difference of nodal metabolic connectivity in NIHL and normal subjects.

	NIHL subjects	Normal subjects	Difference	p	FDR
Left insula					
Degree	50	50	0	0.9170	0
Strength	12.6507	16.7429	4.0922	0.0560	0
Path length	5.1786	4.9956	− 0.1830	0.6710	0
Global efficiency	0.2285	0.2639	0.0354	0.1040	0
Local efficiency	0.4770	0.7286	0.2515	0.0390*	0
Clustering coefficient	0.1547	0.2381	0.0834	0.0320*	0.001
Betweenness centrality	0.0483	0.0230	− 0.0253	0.2680	0
Closeness centrality	0.1931	0.2002	0.0071	0.6830	0
Right insula					
Degree	54	53	− 1	0.8170	0
Strength	12.6095	17.9674	5.3580	0.0210*	0
Path length	5.2495	4.5394	− 0.7101	0.0930	0
Global efficiency	0.2186	0.2816	0.0630	0.0070*	0
Local efficiency	0.4436	0.7489	0.3053	0.0120*	0
Clustering coefficient	0.1424	0.2314	0.0889	0.0130*	0.001
Betweenness centrality	0.0416	0.0498	0.0082	0.8270	0
Closeness centrality	0.1905	0.2203	0.0298	0.0880	0
Right superior temporal gyrus					
Degree	60	64	4	0.4230	0
Strength	11.2629	17.2673	6.0045	0.0110*	0
Path length	5.2881	4.3321	− 0.9559	0.0080*	0
Global efficiency	0.2220	0.2737	0.0517	0.0110*	0
Local efficiency	0.4436	0.6347	0.1911	0.0430*	0
Clustering coefficient	0.1043	0.1664	0.0620	0.0750	0.001
Betweenness centrality	0.0151	0.0490	0.0340	0.2220	0
Closeness centrality	0.1891	0.2308	0.0417	0.0040*	0

*FDR* false discovery rate, *NIHL* noise-induced hearing loss.

*p < 0.05.

## Discussion

The results of this study demonstrated that NIHL subjects had lower cerebral glucose metabolism in both the insulae and right superior temporal gyrus than normal subjects. In terms of metabolic connectivity, both global and nodal parameters were significantly different between the groups.

The insular cortex is covered by the insular opercula of the frontal, parietal, and temporal lobes. It is divided into the anterior and posterior parts by the central insular sulcus^18^. And it is also subdivided into granular, dysgranular, and agranular subdivisions according to the cytoarchitecture^18^. The insular cortex, which is connected to an extensive network of cortical and subcortical brain regions^18^, is known to play a role in visceral sensations, autonomic control, interoception, somatic processing and pain, auditory processing, chemosensory functions, vestibular function, emotional experience, empathy and social cognition, decision making, cognitive functions, and neurological and neuropsychiatric disorders^18,19^. In auditory functions, the insular cortex receives efferent projections from primary auditory, auditory association, and post-auditory cortices^19^. Previous study regarding stroke of insula reported that insular cortex may affect central auditory function^20^. Other study using stereoelectroencephalography demonstrated that the human insula is engaged during auditory deviance detection^21^. The insular cortex is involved in sound detection, non-verbal processing, temporal processing, phonological processing, and visual-auditory integration^22^. Previous studies have shown a correlation between the insula and deafness. Allen et al. reported that the volume of the insula is larger in congenital deaf subjects than in normal subjects^23^. However, other studies have shown no difference in the volume of the insula between deafness and normal subjects^24–26^. It has been reported that subjects with sensorineural hearing loss show hyperperfusion of both insulae and decreased neuronal synchronization between insula subdivisions and other brain regions^24^.

The correlation between hearing loss and cerebral glucose metabolism has been well documented. Previous studies have shown decreased glucose metabolism in the anterior cingulate gyri, superior temporal cortices, and right parahippocampal gyrus in those with post-lingual deafness^9^, decreased glucose metabolism in the right superior temporal gyrus in those with late-onset deafness^10^, decreased glucose metabolism in the right inferior colliculus, and both superior temporal gyrus in those with adult-onset deafness^11^. Previous studies have also demonstrated that cerebral glucose metabolism can predict cochlear implantation outcomes^11,27^. Two studies have shown decreased glucose metabolism in the insula in postlingually deaf subjects and idiopathic sudden sensorineural hearing loss subjects^12,13^, consistent with the results of the present study. NIHL is associated with decreased speech recognition and temporal processing^6^. The insula plays a role in temporal processing and word recognition^22^. Thus, decreased glucose metabolism might reflect the decreased function of both insulae. Decreased glucose metabolism in the superior temporal gyrus of subjects with deafness is well known^9–11^, consistent with the results of this study. The human superior temporal gyrus is known to play a role in hearing, speech, and language^28^. Previous studies using transcranial magnetic stimulation reported that superior temporal gyrus has a role in speech perception^29,30^. The anterior superior temporal gyrus is known to involve both semantic and syntactic processes. The posterior superior temporal gyrus is known to involve integration processes in language or speech, audiovisual integration, biological motion, and face processing^31^. Thus, decreased functions of the superior temporal gyrus might result in decreased glucose metabolism in subjects with NIHL. Decreased glucose metabolism in both insulae in this study was the most different aspect compared to other studies. When NIHL subjects were categorized by WHO grades of hearing impairment, the majority of NIHL subjects had slight or moderate impairment. This difference in the composition of enrolled subjects, such as deafness and slight to moderate hearing loss, might have affected the results of this study. NIHL is a type of sensorineural hearing loss. Previous studies have reported that glucose metabolism and connectivity of the insula are decreased in subjects with sensorineural hearing loss^13,24,32^. These results may reflect the characteristics of sensorineural hearing loss.

In the global measure of metabolic connectivity, the average degree was not different between groups, and the average strength was lower in NIHL subjects than in normal subjects. As degree is the total number of the node’s connections and strength is given by the sum of the weights of all connections linked to the node^33^, this result implies that brain global connectivity between groups is not different and weights of connections in NIHL subjects are lower than in normal subjects. Global efficiency, local efficiency, and mean clustering coefficient were lower in NIHL subjects than in normal subjects. In global measures, global and local efficiency is the average of the global and local efficiencies of all nodes, and the mean clustering coefficient is the average of the nodal clustering coefficients of all nodes^33^. Global efficiency is the inverse of the shortest path from the node to any other node in the network^33^. The closer the nodes are to each other, the more efficient is the transfer of information between them^33^. Thus, decreased global efficiency in NIHL subjects implies lower global network efficiency. Local efficiency is the efficiency of information transfer among the neighbors of a particular node^34^. The clustering coefficient determines the degree of local connectivity of a node with its neighbors^34^. Thus, decreased local efficiency and mean clustering coefficient imply decreased local connectivity and lower local network efficiency. We evaluated nodal parameters in both the insulae and the right superior temporal gyrus. The results were similar to global measures, which showed lower efficiency of both global and local networks and decreased local connectivity. A previous study using MRI reported decreased connectivity of the insula to other brain regions in subjects with sensorineural hearing loss, consistent with this result and concluded that reduced incoming auditory information results in weakened connectivity^24^. Verger et al. reported decreased glucose metabolism in the right superior temporal gyrus; however, they reported that hearing loss subjects showed increased connectivity of the right superior temporal gyrus with the right superior and middle temporal gyri, right precentral frontal and postcentral parietal gyri, right inferior parietal gyrus, cingulum, and both striata^10^. They concluded that the increased connectivity was due to neural plasticity^10^. A study by Ding et al. using MRI reported increased functional connectivity between both the superior temporal gyrus and both the anterior insula and dorsal anterior cingulate cortex in early deafness subjects^35^. Another study using MRI demonstrated that functional connectivity derived from graph-theoretical analysis did not show a significant difference between subjects with sudden unilateral sensorineural hearing loss and healthy controls^36^. Among the previous studies, studies with deafness reported increased connectivity. Also, F-18 FDG PET studies with deafness reported positive correlation between metabolism in both superior temporal gyrus and duration of deafness^9,11^. They concluded that the change of metabolism was due to neural plasticity. Thus, increased connectivity might reflect that the neural plasticity in deafness and decreased connectivity in sensorineural hearing loss subjects without deafness might suggest that neural plasticity was not occurred. Further studies are needed to validate the relationship between neural plasticity and hearing loss.

This study has several limitations. First, the enrolled subjects did not undergo MRI. Different pre-processing processes of PET images and the possibility of hidden brain abnormalities might have affected these results. Second, the enrolled subjects did not undergo a neurocognitive function test. Therefore, neurodegenerative diseases may be present. However, the mean age of enrolled subjects was relatively low and they did not have any clinically diagnosed psychiatric disease or cognitive dysfunction. Therefore, it was highly unlikely that they were affected by presence of psychiatric disease or neurodegenerative disease. Third, DM status, HbA1C, and BMI were known to effect on cerebral glucose metabolism in normal subject. However, only 11 subjects showed either high blood glucose level not less than 126 mg/dL or high HbA1C level not less than 6.5%. And fasting blood glucose, HbA1C, and BMI were not significantly different between NIHL and normal subjects. Thus, we considered that the effect was negligible.

In conclusion, NIHL subjects had lower cerebral glucose metabolism in both the insulae and right superior temporal gyrus than normal subjects. In addition, subjects with NIHL showed decreased metabolic brain connectivity compared to normal subjects. Decreased glucose metabolism and metabolic connectivity in NIHL subject might reflect decreased auditory function. It might be characteristic of sensorineural hearing loss.

## Methods

### Subjects

Data of subjects who had undergone special health examinations, including F-18 FDG PET/CT scans for occupational disease screening in our institute between January 2013 and December 2019 were retrospectively reviewed. The F-18 FDG PET/CT was performed as part of the company's welfare policy. Among them, subjects who were diagnosed with NIHL and those who needed close observation for NIHL were enrolled. The diagnosis of NIHL in these subjects was made by doctors of the Department of Occupational and Environmental Medicine according to the NIHL diagnostic criteria^37^. NIHL was diagnosed when hearing loss was 50 dB or more in the high-pitched range of 4 kHz in pure tone audiometry, and when pure tone average [(0.5 kHz + 1 kHz + 2 kHz)/3] was 30 dB or more. HL should be presumed by noise exposure from occupation history. Subjects who needed close observation for NIHL should have hearing loss not less than 30 dB at 2 kHz, 40 dB at 3 kHz, or 40 dB at 4 kHz. Hearing loss presumed to be due to noise exposure based on occupational history and it must not meet the NIHL diagnostic criteria. Subjects who were diagnosed with NIHL and those who needed close observation for NIHL were assigned to the NIHL subject group in this study. Subjects who were not diagnosed with NIHL by special health examinations were enrolled as normal subjects. Subjects who had a clinically diagnosed neurodegenerative disease, psychiatric disease, or previous cerebral vascular accident were excluded. Subjects who used any neuropsychological medications were also excluded. Both the NIHL and normal subjects were matched for age. As majority of NIHL subjects were males, only male subjects were included. This study was approved by the institutional review board of Samsung Changwon Hospital and informed consent was waived due to retrospective design.

### Imaging protocol

Subjects were intravenously injected with F-18 FDG at a dose of 3.7 MBq/kg (0.1 mCi/kg) body weight. PET/CT scans were performed 60 min after injection using an integrated PET/CT scanner (Discovery 710, GE Healthcare, Waukesha, WI, USA). Before F-18 FDG injection, all subjects were asked to fast for at least 6 h. Fasting blood glucose level was less than 150 mg/dL in all subjects except 3 subjects. As they had uncontrolled diabetes mellitus (DM), the PET/CT scan was performed without any medication. During image acquisition, a CT scan covering the area from the vertex of the skull to the proximal thigh was obtained first for attenuation correction with a slice thickness of 3.75 mm (120 kV). PET data were obtained using a high-resolution whole-body scanner with an axial view field of 15.7 cm. PET images were reconstructed using an iterative algorithm (VUE-Point FX, iteration: 2, subsets: 16) with an image matrix size of 128 × 128.

### Image analysis

Using Amide's Medical Image Data Examiner program, the entire skull area was extracted from each of the original PET and CT scans and converted to a Neuroimaging Informatics Technology Initiative file format. Statistical Parametric Mapping 12 (SPM12; Wellcome Department of Imaging Neuroscience, Institute of Neurology, University College London) was implemented in MATLAB R2020b (MathWorks, Natick, MA) for pre-processing. First, the co-registration of PET images and corresponding CT images was performed for each subject. Second, spatial normalization of PET images into a standard Montreal Neurological Institute (MNI) template was conducted using the deformation field of the CT image. As brain MRI was not performed for enrolled subjects, non-enhanced CT images were used for normalization because a previous study reported a high level of concordance between MRI and CT-based normalization^38^. Third, smoothing of the normalized PET image was performed with a voxel size of 8 × 8 × 8 mm FWHM filter. A two-sample t-test was conducted to compare cerebral glucose metabolism between NIHL and normal subjects using SPM12. Age was included as a covariate. Results were displayed when the uncorrected *p*-value was less than 0.001 and minimum cluster size of 100 contiguous voxels. Coordinates of local maxima were converted from MNI atlas to Talairach space using the Talairach Client v2.4.3.

To evaluate the difference of metabolic connectivity between NIHL subjects and normal subjects, BRAPH–BRain Analysis using graPH theory (http://www.braph.org/), a software package to perform graph theory analysis of the brain connectome was used^33^. Graph analysis was performed by nodes representing brain regions included in a brain atlas and edges representing the relationship between nodes^33^. In this study, the AAL template with 90 ROIs and Pearson correlation coefficients were used to calculate the connectivity. For each group, weighted undirected graphs were built, and non-parametric permutation tests with 1000 permutations were performed to compare the groups. To evaluate the differences between groups in the global network topology, the average degree, average strength, characteristic path length, global efficiency, local efficiency, mean clustering coefficient, transitivity, modularity, assortative coefficient, and small-worldness were calculated. To assess the difference between groups in nodal network topology, the degree, strength, path length, global efficiency, local efficiency, clustering coefficient, betweenness centrality, and closeness centrality were calculaed. Nodal network analysis was evaluated in brain regions showing differences in glucose metabolism between the groups.

### Statistical analysis

Independent t-test was used to compare age, fasting glucose, glycated haemoglobin (HbA1C), BMI (body mass index), pure tone audiometry, and pure tone average between NIHL and normal subjects. Results were considered statistically significant when the p-value was less than 0.05. Data were analyzed using MedCalc^®^ Statistical Software version 20.111 (MedCalc Software Ltd, Ostend, Belgium; https://www.medcalc.org; 2022).

### Ethical approval

All procedures performed in studies involving human participants were in accordance with the ethical standards of the institutional and/or national research committee and with the 1964 Helsinki declaration and its later amendments or comparable ethical standards.

## Data Availability

The datasets used and/or analysed during the current study available from the corresponding author on reasonable request.

## References

[1] Cunningham LL, Tucci DL (2017). Hearing loss in adults. N. Engl. J. Med..

[2] Davis A (2016). Aging and hearing health: The life-course approach. Gerontologist.

[3] Olusanya BO, Davis AC, Hoffman HJ (2019). Hearing loss grades and the international classification of functioning, disability and health. Bull. World Health Organ..

[4] Noise-induced hearing loss. *J. Occup. Environ. Med.* **45**, 579–581 (2003). 10.1097/00043764-200306000-00001 12802210

[5] Natarajan N, Batts S, Stankovic KM (2023). Noise-induced hearing loss. J. Clin. Med..

[6] Le TN, Straatman LV, Lea J, Westerberg B (2017). Current insights in noise-induced hearing loss: A literature review of the underlying mechanism, pathophysiology, asymmetry, and management options. J. Otolaryngol. Head Neck Surg..

[7] Almuhaideb A, Papathanasiou N, Bomanji J (2011). 18F-FDG PET/CT imaging in oncology. Ann. Saudi Med..

[8] Verger A, Guedj E (2018). The renaissance of functional (18)F-FDG PET brain activation imaging. Eur. J. Nucl. Med. Mol. Imaging.

[9] Lee JS (2003). PET evidence of neuroplasticity in adult auditory cortex of postlingual deafness. J. Nucl. Med..

[10] Verger A (2017). Changes of metabolism and functional connectivity in late-onset deafness: Evidence from cerebral (18)F-FDG-PET. Hear. Res..

[11] Han JH, Lee HJ, Kang H, Oh SH, Lee DS (2019). Brain plasticity can predict the cochlear implant outcome in adult-onset deafness. Front. Hum. Neurosci..

[12] Okuda T, Nagamachi S, Ushisako Y, Tono T (2013). Glucose metabolism in the primary auditory cortex of postlingually deaf patients: An FDG-PET study. ORL J. Otorhinolaryngol. Relat. Spec..

[13] Micarelli A (2017). Early cortical metabolic rearrangement related to clinical data in idiopathic sudden sensorineural hearing loss. Hear. Res..

[14] Babaeeghazvini P, Rueda-Delgado LM, Gooijers J, Swinnen SP, Daffertshofer A (2021). Brain structural and functional connectivity: A review of combined works of diffusion magnetic resonance imaging and electro-encephalography. Front. Hum. Neurosci..

[15] Yakushev I, Drzezga A, Habeck C (2017). Metabolic connectivity: Methods and applications. Curr. Opin. Neurol..

[16] Wolak T (2019). Altered functional connectivity in patients with sloping sensorineural hearing loss. Front. Hum. Neurosci..

[17] Speck I (2020). (18)F-FDG PET imaging of the inferior colliculus in asymmetric hearing loss. J. Nucl. Med..

[18] Gogolla N (2017). The insular cortex. Curr. Biol..

[19] Uddin LQ, Nomi JS, Hebert-Seropian B, Ghaziri J, Boucher O (2017). Structure and function of the human insula. J. Clin. Neurophysiol..

[20] Bamiou DE (2006). Auditory temporal processing deficits in patients with insular stroke. Neurology.

[21] Blenkmann AO (2019). Auditory deviance detection in the human insula: An intracranial EEG study. Cortex.

[22] Bamiou DE, Musiek FE, Luxon LM (2003). The insula (Island of Reil) and its role in auditory processing. Literature review. Brain Res. Brain Res. Rev..

[23] Allen JS, Emmorey K, Bruss J, Damasio H (2008). Morphology of the insula in relation to hearing status and sign language experience. J. Neurosci..

[24] Xu XM (2019). Inefficient involvement of insula in sensorineural hearing loss. Front. Neurosci..

[25] Hribar M, Suput D, Carvalho AA, Battelino S, Vovk A (2014). Structural alterations of brain grey and white matter in early deaf adults. Hear. Res..

[26] Shibata DK (2007). Differences in brain structure in deaf persons on MR imaging studied with voxel-based morphometry. AJNR Am. J. Neuroradiol..

[27] Lee HJ (2007). Cortical activity at rest predicts cochlear implantation outcome. Cereb. Cortex.

[28] Howard MA (2000). Auditory cortex on the human posterior superior temporal gyrus. J. Comp. Neurol..

[29] Kennedy-Higgins D, Devlin JT, Nuttall HE, Adank P (2020). The causal role of left and right superior temporal gyri in speech perception in noise: A transcranial magnetic stimulation study. J. Cogn. Neurosci..

[30] Ramos Nunez AI, Yue Q, Pasalar S, Martin RC (2020). The role of left vs. right superior temporal gyrus in speech perception: An fMRI-guided TMS study. Brain Lang..

[31] Friederici AD (2011). The brain basis of language processing: From structure to function. Physiol. Rev..

[32] Luan Y (2019). Dysconnectivity of multiple resting-state networks associated with higher-order functions in sensorineural hearing loss. Front. Neurosci..

[33] Mijalkov M (2017). BRAPH: A graph theory software for the analysis of brain connectivity. PLoS ONE.

[34] Mohan A, De Ridder D, Vanneste S (2016). Graph theoretical analysis of brain connectivity in phantom sound perception. Sci. Rep..

[35] Ding H (2016). Enhanced spontaneous functional connectivity of the superior temporal gyrus in early deafness. Sci. Rep..

[36] Minosse S (2021). Global and local brain connectivity changes associated with sudden unilateral sensorineural hearing loss. NMR Biomed..

[37] Kim KS (2010). Occupational hearing loss in Korea. J. Korean Med. Sci..

[38] Presotto L (2018). Low-dose CT for the spatial normalization of PET images: A validation procedure for amyloid-PET semi-quantification. Neuroimage Clin..

